# Supplementary material for: Examining the impact of ICU population interaction structure on modeled colonization dynamics of Staphylococcus aureus
Source: PLoS Comput Biol. 2022 Jul 25;18(7):e1010352. doi: 10.1371/journal.pcbi.1010352 (PMC9352208; doi:10.1371/journal.pcbi.1010352)
Supplement: S1 Table — (DOCX) [file pcbi.1010352.s001.docx]

**Population Structure Drives Differential Methicillin-resistant *Staphylococcus aureus* Colonization Dynamics**

**Supplemental Material**

**Table S1** Transitions and Equations for the Single Staff Type (SST) Model of MRSA Acquisition

| Process | Event | Transition | Equation |
| --- | --- | --- | --- |
| MRSA Acquisition & Transmission | Staff Contaminated | S_U_ to S_C_ | $\rho\sigma S_{U}\frac{P_{C}}{(P_{C}+P_{U})}$ |
|  | Patient Colonized | P_U_ to P_C_ | $\rho P_{U}\frac{S_{C}}{(S_{C}+S_{U})}$ |
| MRSA Decolonization | Natural De-colonization | P_C_ to P_U_ | μ$P_{C}$ |
| Hand Hygiene and Decontamination | Hand Decontamination | S_C_ to S_U_ | $\iota S_{C}$ |
|  | PPE Change | S_C_ to S_U_ | $\tau S_{C}\frac{P_{C}}{P_{C}+P_{U}}$ |
| Patient Admissions and Discharge | P_U_ Discharge to P_U_ Admission* |  | $\theta\nu_{U}P_{U}$ |
|  | P_U_ Discharge to P_C_ Admission* |  | $\theta\nu_{C}P_{U}$ |
|  | P_C_ Discharge to P_U_ Admission* |  | $\theta\nu_{U}P_{C}$ |
|  | P_C_ Discharge to P_C_ Admission* |  | $\theta\nu_{C}P_{C}$ |

* Note that patient discharge to patient admissions are not true “transitions” of a single individual, but rather the instantaneous replacement of a discharged patient with a newly admitted patient to maintain a steady population state. See 37 for details.
