# Supplementary material for: Examining the impact of ICU population interaction structure on modeled colonization dynamics of Staphylococcus aureus
Source: PLoS Comput Biol. 2022 Jul 25;18(7):e1010352. doi: 10.1371/journal.pcbi.1010352 (PMC9352208; doi:10.1371/journal.pcbi.1010352)
Supplement: S2 Table — (DOCX) [file pcbi.1010352.s002.docx]

**Population Structure Drives Differential Methicillin-resistant *Staphylococcus aureus* Colonization Dynamics**

**Supplemental Material**

**Table S2** Transitions and Equations for the Nurse-MD Model of MRSA Acquisition

| Process | Event | Transition | Equation |
| --- | --- | --- | --- |
| MRSA Acquisition & Transmission | Nurse Contaminated | N_U_ to N_C_ | $\rho_{N}{\sigma N}_{U}\frac{P_{C}}{(P_{C}+P_{U})}$ |
|  | Doctor Contaminated | D_U_ to D_C_ | $\rho_{D}{\sigma D}_{U}\frac{P_{C}}{(P_{C}+P_{U})}$ |
|  | Patient Colonized (Nurse Contact) | P_U_ to P_C_ | $\rho_{N}P_{U}\frac{N_{C}}{(N_{C}+N_{U})}$ |
|  | Patient Colonized (Physician Contact) | P_U_ to P_C_ | $\rho_{D}P_{U}\frac{D_{C}}{(D_{C}+D_{U})}$ |
| MRSA Decolonization | Natural De-colonization | P_C_ to P_U_ | μ$P_{C}$ |
| Hand Hygiene and Decontamination | Nurse Hand Decontamination | N_C_ to N_U_ | ${\iota_{N}N}_{C}$ |
|  | Physician Hand Decontamination | D_C_ to D_U_ | ${\iota_{D}D}_{C}$ |
|  | Nurse PPE Change | N_C_ to N_U_ | ${\tau_{N}N}_{C}\frac{P_{C}}{(P_{C}+P_{U})}$ |
|  | Physician PPE Change | D_C_ to D_U_ | ${\tau_{D}D}_{C}\frac{P_{C}}{(P_{C}+P_{U})}$ |
| Patient Admissions and Discharge | P_U_ Discharge to P_U_ Admission* |  | $\theta\nu_{U}P_{U}$ |
|  | P_U_ Discharge to P_C_ Admission* |  | $\theta\nu_{C}P_{U}$ |
|  | P_C_ Discharge to P_U_ Admission* |  | $\theta\nu_{U}P_{C}$ |
|  | P_C_ Discharge to P_C_ Admission* |  | $\theta\nu_{C}P_{C}$ |

* Note that patient discharge to patient admissions are not true “transitions” of a single individual, but rather the instantaneous replacement of a discharged patient with a newly admitted patient to maintain a steady population state. See 37 for details.
