# Supplementary material for: Examining the impact of ICU population interaction structure on modeled colonization dynamics of Staphylococcus aureus
Source: PLoS Comput Biol. 2022 Jul 25;18(7):e1010352. doi: 10.1371/journal.pcbi.1010352 (PMC9352208; doi:10.1371/journal.pcbi.1010352)
Supplement: S3 Table — (DOCX) [file pcbi.1010352.s003.docx]

**Population Structure Drives Differential Methicillin-resistant *Staphylococcus aureus* Colonization Dynamics**

**Supplemental Material**

**Table S3** Transitions and Equations for the Metapopulation Model of MRSA Acquisition

| Process | Event | Transition | Equation |
| --- | --- | --- | --- |
| MRSA Acquisition & Transmission | Nurse Contaminated (Assigned Patient) | N_U,i_ to N_C,i_ | $\rho_{N}{\sigma N}_{Ui}\frac{P_{Ci}}{\left( P_{Ci}+P_{Ui} \right)} ; i=1\ldots6$ |
|  | Nurse Contaminated (Unassigned Patient) |  | $\rho_{N}{\sigma N}_{Ui}\frac{P_{Cj}}{(P_{Cj}+P_{Uj})} {[(1- )}/{5]} ; i=1\ldots6, j=1\ldots6, j\neq i$ |
|  | Physician Contaminated | D_U_ to D_C_ | $\rho_{D}{\sigma D}_{U}\frac{\begin{aligned} \sum_{\begin{aligned} i=1 \end{aligned}}^{6} \begin{aligned} P_{Ci} \end{aligned} \end{aligned}}{\begin{aligned} \sum_{i=1}^{6} \begin{aligned} \left( P_{Ci}+P_{Ui} \right) \end{aligned} \end{aligned}}$ |
|  | Patient Colonized (Assigned Nurse Contact) | P_U,i_ to P_C,i_ | $\rho_{N}P_{Ui}\frac{N_{Ci}}{(N_{Ci}+N_{Ui})} ; i=1\ldots6$ |
|  | Patient Colonized (Unassigned Nurse Contact) |  | $\rho_{N}P_{Ui}\frac{N_{Cj}}{(N_{Cj}+N_{Uj})} {[(1- )}/{5]} ; i=1\ldots6, j=1\ldots6, j\neq i$ |
|  | Patient Colonized (Physician Contact) | P_U.i_ to P_C.i_ | $\rho_{D}P_{Ui}\frac{D_{C}}{(D_{C}+D_{U})} ; i=1\ldots6$ |
| MRSA Decolonization | Natural De-colonization | P_C,i_ to P_U,i_ | μ$P_{Ci} ; i=1\ldots6$ |
| Hand Hygiene and Decontamination | Nurse Hand Decontamination (Assigned Patient) | N_C,i_ to N_U,i_ | ${\iota_{N}N}_{Ci} ; i=1\ldots6$ |
|  | Physician Hand Decontamination | D_C_ to D_U_ | ${\iota_{D}D}_{C}$ |
|  | Nurse PPE Change (Assigned Patient) | N_C,i_ to N_U,i_ | ${\tau_{N}N}_{Ci}\frac{P_{Ci}}{\left( P_{Ci}+P_{Ui} \right)} ; i=1\ldots6$ |
|  | Nurse PPE Change (Unassigned Patient) |  | ${\tau_{N}N}_{Ci}\frac{P_{Cj}}{(P_{Cj}+P_{Uj})} {[(1- )}/{\begin{aligned} 5] ; i=1\ldots6, j=1\ldots6, j\neq i \end{aligned}}$ |
|  | Physician PPE Change | D_C,i_ to D_U,i_ | ${\tau_{D}D}_{C}\frac{\begin{aligned} \sum_{\begin{aligned} i=1 \end{aligned}}^{6} \begin{aligned} P_{Ci} \end{aligned} \end{aligned}}{\begin{aligned} \sum_{i=1}^{6} \begin{aligned} \left( P_{Ci}+P_{Ui} \right) \end{aligned} \end{aligned}}$ |
| Patient Admissions and Discharge | P_U,i_ Discharge to P_U,i_ Admission* |  | $\theta\nu_{U}P_{Ui} ; i=1\ldots6$ |
|  | P_U,i_ Discharge to P_C,i_ Admission* |  | $\theta\nu_{C}P_{Ui} ; i=1\ldots6$ |
|  | P_C,i_ Discharge to P_U,i_ Admission* |  | $\theta\nu_{U}P_{Ci} ; i=1\ldots6$ |
|  | P_C,i_ Discharge to P_C,i_ Admission* |  | $\theta\nu_{C}P_{Ci} ; i=1\ldots6$ |

* Note that patient discharge to patient admissions are not true “transitions” of a single individual, but rather the instantaneous replacement of a discharged patient with a newly admitted patient to maintain a steady population state. See 37 for details.
