# Supplementary material for: Examining the impact of ICU population interaction structure on modeled colonization dynamics of Staphylococcus aureus
Source: PLoS Comput Biol. 2022 Jul 25;18(7):e1010352. doi: 10.1371/journal.pcbi.1010352 (PMC9352208; doi:10.1371/journal.pcbi.1010352)
Supplement: Attachment — Submitted filename: Response.docx [file pcbi.1010352.s004.docx]

October 11, 2021

Drs. Althouse and De Boer,

Please find enclosed a revision to PCOMBIOL-D-19-01296, renamed ‘Examining the Impact of ICU Population Interaction Structure on Modeled Colonization Dynamics of Staphylococcus aureus’. We apologize for the time it has taken for this revision – the ongoing COVID-19 pandemic has necessitated a reshuffling of priorities, and we wanted to make sure we had fully considered and addressed the reviewer’s comments before submitting this revision.

Below is a point-by-point response to the reviewer comments. We believe that these have substantially improved the manuscript, and in broad terms have made it more clear that the substantive thrust of the paper is not proposing that our model is itself novel, but that it represents an important element of the evidence base confirming the necessity of using more complex models, including network and agent-based models, in modeling healthcare associated infections.

Thank you again for your consideration, and the opportunity to revise this manuscript.

Regards,

Eric Lofgren MSPH, PhD

Assistant Professor

Paul G. Allen School for Global Health

Washington State University

*Reviewer #1: In the paper by Mietchen et al, the authors build upon a stochastic compartmental model to examine MRSA transmission in an ICU. They compare and contrast random vs. non-random mixing approaches in patient care and conclude that random mixing assumptions may be inappropriate. I congratulate the authors on developing a computational model examining an important topic (which I recognize takes many hours to do) yet I find their conclusion somewhat underwhelming as, in fact, this has been a known limitation in the epidemiology modeling field for some time. We have seen the rise of approaches that relax or omit this assumption over the past decade, but these other approaches are not mentioned in the manuscript. This ties in with my main concern: the background and discussion do not acknowledge the wealth of work done in modeling infectious diseases that do not assume a random mixing approach. I also have the following comments for the authors to consider to additionally improve this work.*

We thank the reviewer for this comment – it is apparent from it that we have failed to communicate the primary intention for the analysis, which was done in acknowledgement of the wealth of work that doesn’t assume random mixing. It has been the experience of the authors that network and agent-based models for healthcare-associated infections are extremely well represented – and indeed, all five original sites of the CDC’s Modeling Infectious Diseases in Healthcare Network were using these models, including our own. However, in most cases there was a distinct lack of any attempt to quantify if the additional effort was worth it in terms of a change in the results.

It is our goal, with this manuscript, to help provide that example – showing that the step from a simpler model to a more complex one that is, while not on the level of a network or agent-based model in terms of how it represents contact, considerably more structure is, in a number of cases, quite impactful.

We have also noted that many models of system-level policies, including those by the CDC, Lee et al.’s 2011 paper on MRSA in Orange County and the many papers that follow it, etc. have accepted the use of simpler compartmental models for within hospital transmission, meaning models like the SST model remain in widespread use, albeit often in a form that’s difficult to identify. We wanted to examine the impact of that.

*Please define consistent care teams: Are the authors referring to same providers, roles, or something else? What about shift changes?*

This has been defined.

*“grouping particular types of patients together”: Please use the term “cohorting”. This is the standard term in hospital infection prevention.*

We originally wanted to avoid cohorting, as this sort of grouping may also include sorting patients for ease of rounding or other administrative decisions rather than just cohorting, but we have added that as one of the possible subtypes.

*The lit review seems to have missed some recent papers that examine how the built hospital environment and patient care networks impact MRSA transmission in the ICU. Some of these papers are based in the neonatal ICU, but have modeling implications for the adult ICU as well.*

Several references have been added.

*Re: violating the independence assumption of many statistical techniques. I agree with the authors, and thus researchers use a variety of models with correlated errors to account for this occurrence. Would acknowledge that there are techniques that do not depend on the independence assumption.*

*Re: limited or no control groups. But what about multicenter studies that either do a site-level randomization, or purely observational work? While differences between sites exist, the process of delivering care in an ICU is fairly standardized in the U.S.*

In the extensive reworking of the introduction to better reflect the manuscript’s intend, these sections have been removed.

*The authors state that “widely used models generally assume random mixing between healthcare workers and patients,” but there are no cites to back this claim up. This may have been true a decade or so ago, but use of network models relax the random mixing approach and in fact are now expected and move beyond compartmental modeling approaches. This is why in the epidemiology literature we see the increase in ABMs and network models, yet there is no acknowledgement of these in the paper. Examples exist in both the healthcare and non-healthcare environments.*

It is our experience that these models remain relatively common, either stand-alone or incorporated as a sub-model in more complicated agent-based models. Further, while there is definitely a trend toward these models, the authors have found the justifications of this to be primarily heuristic, and we seek to provide a quantification of the potential impact of these decisions.

*Why include the SST model? This is not realistic. In fact, the majority of care in an ICU is provided by the nurse and not the physicians. Hand hygiene can also be differentially modeled by provider role.*

While we agree, we find models that incorporate healthcare workers as a general class (or SEIR-type models that obfuscate their role into a single parameter) are still relatively common. While We agree that the SST model is not realistic, those who would argue for parsimonious models might prefer it’s analytical tractability, and so it seemed reasonable to include it as an example, and examine where it might have room as an acceptable approximation.

*More detail regarding the setting is needed (brought in from the referenced publication). Is this meant to reflect an actual ICU or a hypothetical ICU? What type of ICU? How many admissions per year? Especially since the authors rely on parameter estimates from [17] that combines med/surg ICUs, these could be very different in terms of risk for organism colonization and infection.*

More detail has been added. Note however that this is meant to represent a hypothetical ICU.

*Re: MRSA colonization/detection assumption. Does this ICU carry out some kind of surveillance for MRSA colonization? Further, if an individual is colonized with MRSA, is a decolonization regimen performed (aside from the “natural” decolonization employed)? These are important considerations to accurately model MRSA transmission.*

We have added text detailing our assumptions here. There is no actively modeled surveillance or decolonization regimen performed – while we agree that these would be employed (and indeed, the meta-population model has been used to estimate the effectiveness of decolonization procedures) any ongoing interventions would necessarily drive the estimated colonizations for all models toward zero, obfuscating the differences induced by model structure vs. the intervention. As such, we have not included any interventions to make the structural impact as clear as possible.

*Perhaps the authors can consider stratifying potential for contamination/transmission based on types of interactions with the patients. For example, there are a lot of routine, more mundane activities that confer lower risk, and fewer, invasive activities that confer higher risk. This would add an interesting and novel aspect to this work.*

We are currently working on a model that does this – however, the added complexity there also masks some of the impact of the structural decisions examined in this manuscript.

*While the model [appears to be] calibrated, was it also validated to ensure it reflects some kind of real ICU? This will make the findings more relevant for infection preventionists. Especially because in the Discussion the authors state that these models are useful “only if they can represent the population and transmission dynamics of a hospital.” This goes back to my earlier comment to better describe the setting.*

The model, its output, and its results have been discussed with a number of infection prevention teams, who have found the dynamics represented to be satisfactory.

*I applaud the authors for releasing their analytic code and data. More research groups should do this.*

Thank you.

*Figure 4/number of MRSA acquisitions: Please include a denominator, such as patient-days, when presenting incidence. This will make the findings comparable.*

Figure 4 has been reworked to reflect acquisitions per 1,000 patient-days.

*Would be nice to include estimates of the proportion of MRSA transmissions due to the provider role under each scenario.*

A paper examining this over a range of staffing ratios is currently in progress.

*Can the authors provide an intuitive interpretation of gamma=0.4? How does this finding potentially impact patient care dynamics in an ICU?*

In the conclusions we have converted this to a percentage, and provided several informal estimates from infection control colleagues as to where their ICUs are.

*The discussion needs to be expounded to compare and contrast to other work in this area, as well as provide recommendations for how the findings are useful for infection control in an ICU setting.*

*The authors state that the “finding has broad implications for staffing levels and hospital policy." Such as ???*

We have attempted to include more discussion regarding these points, but also have attempted to reemphasize that there is a substantial methodological thrust to the manuscript.

*The authors note the lack of non-nurse/MD personnel and visitors in their model. How does this omission potentially impact findings?*

A brief discussion of this has been added.

*How does the MRSA colonization/detection assumption impact findings? Especially because this is an important assumption and not reflective of how MRSA is actually detected. Suggest the authors bring in a surveillance aspect to this, and (possibly) decolonization if they use this in their ICU.*

This is an excellent question. An extended discussion of the impact of detection and decolonization in the metapopulation model can be found here: https://jamanetwork.com/journals/jamanetworkopen/fullarticle/2776969

*Patient cohorting is mentioned in the introduction, but not returned to in the Discussion or elsewhere. Another opportunity to add an interesting angle to this work would be to compare and contrast cohorting and isolation of MRSA carriers.*

The authors thank the reviewer for this suggestion – we are concerned that this would somewhat obscure the structural results of this manuscript, and would like to examine that question in a broader context comparing pathogens that are frequently introduced to the ICU (i.e. MRSA) with those that are more sporadic.

*Reviewer #2: This paper uses a modeling-based approach to compare several different structures of hospital ICU population interactions and how that affects MRSA acquisition rates.  The goal seems to be to inform future models of MRSA acquisition within ICU settings, noting that simpler models overestimate both MRSA acquisition and the potential effects of interventions.  This seems important and the importance of the findings could be highlighted more in the introduction.*

*The science in this work appears very well thought out, but a few improvements in the writing could make it easier for the reader to follow and understand earlier on in the paper.*

We thank Reviewer 2 for this comment, and hope they find the reworking of the introduction to make this intention clearer.

*short title includes “ICU”, main title should include “ICU” too*

The main title has been changed.

*the term “Population structure” doesn’t seem quite right—perhaps “population interaction structure” is more accurate?*

This has been changed as well.

*whenever feasible, it would be helpful for the reader to write out what the parameter is measuring, rather than just the parameter letter*

We have included this where possible in the manuscript

*it would be helpful to have a more general description of what the models measure and what they take into account earlier on (rather than just the population interaction structures)*

We have added more detail.

*it would also be helpful to label whatever parameters you could on the diagrams*

We have gone back and forth on this point, and have found that adding parameter labels to the diagrams tends to make them less, rather than more, legible.

*at line 306, is the word contact correct?  "The altered contact patterns in the Meta-population model thus needs substantially higher per-contact colonization probabilities to sustain the same level of contact.”*

No, this should be “same level of colonization”, and this has been corrected.

*Figure 5: it would be easier to compare the 4 panels if the parameters were either in the same order or color-coded*

The parameters are in the same order between all figures – however, because of the split from a single “HCW” staff type to nurses and doctors specifically, Panel A inherently has fewer parameters. When they are split however, we attempt to keep them in the same order.
