# Supplementary material for: Examining the impact of ICU population interaction structure on modeled colonization dynamics of Staphylococcus aureus
Source: PLoS Comput Biol. 2022 Jul 25;18(7):e1010352. doi: 10.1371/journal.pcbi.1010352 (PMC9352208; doi:10.1371/journal.pcbi.1010352)
Supplement: Attachment — Submitted filename: PLOS_CompBio Response Letter.docx [file pcbi.1010352.s005.docx]

Feb. 7, 2022

Drs. Althouse and De Boer,

Please find enclosed two versions of the revised manuscript PCOMPBIOL-D-19-01296R1 titled, ‘Examining the Impact of ICU Population Interaction Structure on Modeled Colonization Dynamics of *Staphylococcus aureus*’. One version (document name) containing tracked changes denoting changes made to the text, and another version (document name) containing the same changes but without the changed documented.

Below you will find a detailed list of the responses to the reviewer’s comments containing a description of the changes made within the manuscript. We feel that these changes are significant towards the improvement of the manuscript. Clarifying and redirecting the background information to support the main point of the research, which is to consider using simpler models with complex structures as a generalizable method for studying the spread of HAIs in ICU or hospital settings. In light of the increasing use of network and agent-based models for hospital epidemiology, we believe this remains an important evaluation of the structural sensitivity of the models we use.

Thank you again for the opportunity to revise this manuscript and considering it for publication.

Best Regards,

Eric T. Lofgren, MSPH PhD

Assistant Professor

Paul G. Allen School for Global Health

Washington State University

*Reviewer #2: On re-review of this manuscript by Mietchen et al, I do find that I understand it better, but largely because of their responses to reviewers. More of these responses should make it into the paper.*

*This is good work that will help inform when certain models are appropriate to use, but the writing needs a lot of work. The introduction and discussion sections especially need to be more focused and set up the question better. And relevant models, such as from the 5 CDC sites, need to be cited. Much more citation of the modeling literature is needed—especially in the discussion section, which currently has zero citations.*

We thank the reviewer for this comment, and hope they find this reworked and revised version to both the introduction and discussion sections satisfactory. The focus of the background and main question of the paper has been reorganized to hopefully be more apparent, and more contextual citations have been added.

*General*

- *The main point of sentences should more often be at the beginning of the sentence so it’s easier to follow.*
- *For future versions, please use track changes correctly.*
- *It appears that you just cut everything and pasted—making it appear that everything was entirely re-written, which is not the case (the 1st sentence of the intro is the same.*
- *The writing throughout could be much more concise.*

We hope the sentence structure and writing revisions are more succinct, concise, and easier to follow.

We have used the compare tool in Microsoft Word to provide the track changes version, as this allows for the presentation of the changes from multiple authors as a single omnibus version. Overall, we believe this accurately represents the changes involved. There are one or two exceptions, where it appears the software is representing a rewrite of a section as a wholesale deletion and rewrite. We apologize in advance for this ambiguity.

*Abstract*

- *The “background” section isn’t actually background or justification for why you’re comparing these models—you need to set up your story. The author summary did a better job of this.*

We hope the changes and reworking of the background section provides a clearer justification.

*Introduction*

- *The introduction currently focuses too much and too early on the specifics of MRSA and infection prevention. It needs to highlight much more and earlier on, the importance of models in healthcare settings, the desire for simple models, and the question of whether simple models are appropriate.*
- *The specific details of MRSA aren’t as important and can be briefly summarized and cited.*

The entire introduction section has been reworked, considering the entirety of the suggestions mentioned here. Better justification of the main question of ‘Can we use a more complex model that is not agent-based or a network model to approximate MRSA acquisitions in an ICU?” In addition, MRSA background and justification has been kept to a minimum and most of it was realized to be better suited for discussion rather than introduction.

*Methods*

- *Model Structure section is too wordy. Can be shortened substantially.*
- *Cite Table 1 earlier on in your description of the model structures, and also cite the supplement with the equations.*

We have attempted to shorten the Model Structure section as much as possible without harming clarity.

All suggested citations and references to Table 1 and the equations in the supplemental documentation were added to the model structure section.

*Figures*

- *Note in all figure legends that arrows represent parameters found in table 1, and that equations are found in supplement.*

All suggested citations and references to Table 1 and the equations in the supplemental documentation were added to each figure legend.
