# Supplementary material for: Examining the impact of ICU population interaction structure on modeled colonization dynamics of Staphylococcus aureus
Source: PLoS Comput Biol. 2022 Jul 25;18(7):e1010352. doi: 10.1371/journal.pcbi.1010352 (PMC9352208; doi:10.1371/journal.pcbi.1010352)
Supplement: Attachment — Submitted filename: re-re-review.docx [file pcbi.1010352.s006.docx]

The authors have added the necessary information to the introduction and discussion. I think this is important work, worth publishing, however it reads a bit clunkily. Here are some suggestions:

1. Figures 1- 3 should be combined, and the hybrid model should be included, as shown (right). I will now refer to Figure 1A-D as I have edited.

Figure 1. Compartmental models of methicillin-resistant Staphylococcus aureus (MRSA) acquisition. Patients and staff are classified as (un)colonized or (un)contaminated (U and C on diagrams), respictively. Solid arrows indicate transition states, while dashed arrows indicate routes of MRSA transmission (transition parameters and equations are Table 1 and S1, respectively). A)Single staff type model, B) Nurse-MD model, C) metapopulation model in which nurses are assigned patient groups, and D) a hybrid model where nurses only spend a fraction of their time in their assigned patient groups and otherwise see patients at random.

a) A suggegested revised caption is below the figure.

2. Figure 1A
   a) should not have the nurse icon since it is specifically a *single staff model* (mabe use a color in between the orange and red, or keep the orange and use a red border? Either get rid of the icon or use both nurse and md icons?
   b) Missing the arrow from P_c to P_u
3. Figure 1B is also missing the arrow from P_c to P_u
4. Table 1
   a) Include gamma
5. Supplemental tables of equations:
   a) There should only be one row/equation per transistion (instead of 1 per term)
6. Figure 5
   a) Add parameter descriptions to the plot, not just the Greek letters
   b) Change the x-axis legend to add “per 1% change in parameter value”
   c) Consider either arranging parameters 1) in the same order on all plots, or 2) in order of the biggest to smallest impact, or 3) could color-code and have one legend for all 4 panels, to save text space
   d) Since you are using >0.20 as a cutoff to indicate “large changes”, consider adding an indicator/asterisk to bars with a change of >0.2.
7. Figure 6
   a) Y-axis legend: add units (# of MRSA acquisitions per XXXX)
   b) X-axis legend: add gamma description
8. General:
   a) The point of this analysis is to compare models, so none of them are really “alternative”.
   b) Figures inset at the end of the paper are not the same as those attached (there is an error in Figure 1—all U’s, no C’s)
   c) Models don’t have “experiences” (line 343).
   d) Are the units in figures 4 and 6 (MRSA acquisitions) different? They should be comparable (and units should always be noted).
   e) In general, the writing is not conscise and while passable as is, would befefit from tightening it up—perhaps help from a writing lab?
   f) Please consider revising tables with the layouts below, to improve readability and decrease frustration with trying to keep track of a long list of parameter values.

Suggested format for table 1:

| Description | | |  |  |
| --- | --- | --- | --- | --- |
| General | Units | Specific | Parameter | Value |
| Contact rate | Rate  # of direct care tasks per hour | Pts & HCWs | Rho | 4.154 |
|  |  | Pts & nurses | Rho_N | 3.973 |
|  |  | Pts & physician | Rho_D | 0.181 |
| Transmission  (contamination or colonization) | Probability (0-1) | Pts to HCW (single contact) | sigma | 0.054 |
|  |  | HCW to Pt – SST model | Psi_SST | 0.1494 (fitted) |
|  |  | HCW to Pt – Nurse-MD model | Psi_Nurs-MD | 0.1660 (fitted) |
|  |  | HCW to Pt – metapop model | Psi_Metapop | 0.4481 (fitted) |
| Discharge | Rate  # discharges per day | NA | Theta | 0.228 (1/4.39) |
| Colonized admissions | Proportion (0-1) | Patient colonized with MRSA | Nu_c | 0.0779 |
|  |  | Patient uncolonized | Nu_u, or 1 – Nu_c | 0.9221 |
| Hand Decontaminations  (Direct care tasks x hand hygiene compliance x efficacy) | Rate  (# effective decons per hour) | HCW | Iota | Why are these numbers in your table not multiplied by the contact rates (rho) above???? |
|  |  | Nurse | Iota_N |  |
|  |  | Physician | Iota_D |  |
| Gown changes  (number of visits * compliance) | Rate  # gown changes per hour | HCW | Tau |  |
|  |  | Nurse | Tau_N |  |
|  |  | Physician | Tau_D |  |
| Natural de-colonization | Rate  Decolonizations per day | NA | Mu | 0.05 |
| Amount of time nurses stay in their assigned group | Proportion (1/6-1) |  | gamma | 1/6 = Nurse-MD  1 = metapopulation |

**Suggested format for Table S1**

| Description | Specific | Transition | Equation | Narrative Equation  [in addition to populations interacting] |
| --- | --- | --- | --- | --- |
| MRSA acquisition | Staff contaminated | S_U_ to S_C_ | $\rho\sigma S_{U}\frac{P_{C}}{(P_{C}+P_{U})}$ | Contact rate * contamination probability |
|  | Patient colonized | P_U_ to P_C_ | $\rho\psi P_{U}\frac{S_{C}}{(S_{C}+S_{U})}$ | Contact rate * colonization probability |
| MRSA loss | Staff de-contaminated | S_C_ to S_U_ | $\iota S_{C}$ + $\tau S_{C}\frac{P_{C}}{P_{C}+P_{U}}$ | Hand decontaminations + gown changes |
|  | Patient de-colonized | P_C_ to P_U_ | μ$P_{C}$ | Natural decolonization rate |
| Patient discharges to admissions  (not individual level transitions) | P_U_ to P_U_ | These aren’t exactly transitions like all the others, as in—it's not the same individual being discharged and admitted…(can you note this somehow?) | $\theta\nu_{U}P_{U}$ | Discharge rate * proportion (un)colonized patients admitted |
|  | P_U_ to P_C_ |  | $\theta\nu_{C}P_{U}$ |  |
|  | P_C_ to P_U_ |  | $\theta\nu_{U}P_{C}$ |  |
|  | P_C_ to P_C_ |  | $\theta\nu_{C}P_{C}$ |  |
