# Supplementary material for: Examining the impact of ICU population interaction structure on modeled colonization dynamics of Staphylococcus aureus
Source: PLoS Comput Biol. 2022 Jul 25;18(7):e1010352. doi: 10.1371/journal.pcbi.1010352 (PMC9352208; doi:10.1371/journal.pcbi.1010352)
Supplement: Attachment — Submitted filename: PLOS_CompBio Response Letter.docx [file pcbi.1010352.s007.docx]

May 19, 2022

Drs. Althouse and De Boer,

Please find enclosed two versions of the revised manuscript PCOMPBIOL-D-19-01296R2 titled, ‘Examining the Impact of ICU Population Interaction Structure on Modeled Colonization Dynamics of *Staphylococcus aureus*’. One version containing tracked changes denoting changes made to the text, and the other a clean copy of the revision.

Below you will also find a detailed list of the responses to Reviewer 2’s comments, containing a description of the changes made within the manuscript – or our reasoning for leaving the manuscript as-is. We believe these changes should satisfy the reviewer’s desire for increased clarity.

Thank you again for the opportunity to revise this manuscript and considering it for publication.

Best Regards,

Eric T. Lofgren, MSPH PhD

Assistant Professor

Paul G. Allen School for Global Health

Washington State University

*Reviewer 2:*

*The authors have added the necessary information to the introduction and discussion. I think this is important work, worth publishing, however it reads a bit clunkily.*

We thank the reviewer for this comment, and we hope they find the included revisions improve the clarity of the work.

*Figures 1- 3 should be combined, and the hybrid model should be included, as shown (right). I will now refer to Figure 1A-D as I have edited. A suggested revised caption is below the figure.*

*Figure 1A*

*a)	should not have the nurse icon since it is specifically a single staff model (mabe use a color in between the orange and red, or keep the orange and use a red border? Either get rid of the icon or use both nurse and md icons?*

*b)	Missing the arrow from P_c to P_u*

*Figure 1B is also missing the arrow from P_c to P_u*

This revision to Figure 1 has been made. Other figures have been renumbered accordingly.

*Table 1*

*c)	Include gamma*

Gamma has been added to table 1.

*Supplemental tables of equations:*

*d)	There should only be one row/equation per transistion (instead of 1 per term)*

We disagree with this – the formatting was chosen here for two reasons:

1) Each term represents a different biological or epidemiological mechanism and keeping them separate acknowledges this.
2) This approach means the presentation in the table corresponds more closely to the implementation in the code.

Given the relative lack of urgency for brevity in supplemental material, we believe this expanded version’s improved clarity is valuable. Given that the transition terms are not actually systems of equations, we feel the current format is appropriate.

*Figure 5*

*e)	Add parameter descriptions to the plot, not just the Greek letters*

*f)	Change the x-axis legend to add “per 1% change in parameter value”*

*g)	Consider either arranging parameters 1) in the same order on all plots, or 2) in order of the biggest to smallest impact, or 3) could color-code and have one legend for all 4 panels, to save text space*

*h)	Since you are using >0.20 as a cutoff to indicate “large changes”, consider adding an indicator/asterisk to bars with a change of >0.2.*

Figure 3 nee 5 has been revised in several ways. We have changed the x-axis legend, and we have included a grey boundary marker to indicate that cutoff value. We explored using parameter descriptions, however even with a single-panel figure this proved far too cluttered. Finally, we note that the parameters are in the same order on all plots – the reason they differ is that some parameters (e.g. tau and iota) expand into two parameters for some of the models. We believe the current method of having these show up in place in the more complex figures is clearer than any other approach we have considered. The legend has been revised to note the boundary lines.

*Figure 6*

*i)	Y-axis legend: add units (# of MRSA acquisitions per XXXX)*

*j)	X-axis legend: add gamma description*

Figure 4 nee 6 has been revised to add units and a description of gamma.

*The point of this analysis is to compare models, so none of them are really “alternative”.*

Given the dominance of the classic Ross-McDonald style model in terms of compartmental model representations of healthcare environments, we believe these models are accurately referred to as alternatives, as “alternative” simply indicates another possibility. However, at least one instance of this has been replaced with more specificity.

*Figures inset at the end of the paper are not the same as those attached (there is an error in Figure 1—all U’s, no C’s)*

This was the result of a drift between the EPS versions in the submitted paper and the in-line PDF versions. This has been fixed.

*Models don’t have “experiences” (line 343).*

This sentence has been revised.

Are the units in figures 4 and 6 (MRSA acquisitions) different? They should be comparable (and units should always be noted).

This has been resolved. Figure 4 nee 6 has been changed to rates rather than counts to be comparable, and the units noted.

*In general, the writing is not conscise and while passable as is, would befefit from tightening it up—perhaps help from a writing lab?*

The current manuscript has now been proofed and reviewed by an additional individual. We hope the reviewer will find this has resulted in increased clarity.

*Please consider revising tables with the layouts below, to improve readability and decrease frustration with trying to keep track of a long list of parameter values.*

The supplemental table has been revised. We have not however revised Table 1 – the external reader mentioned above expressed a preference for the current format of the table in terms of readability, and we were unable to retain both the reviewers preferred formatting and the longer form description of the parameters and the source of those values while maintaining both readability and keeping the table to a single page.

To address the question posed in your table, *“Why are these numbers in your table not multiplied by the contact rates (rho) above????”*, this is because the contact rates for rho are for each *patient* not each healthcare provider. The transformation is straightforward, however as you note, the table is already relatively complicated, and we wish to avoid this.

Further, this format has been used in several previous papers, so we are electing to continue to use it for comparability. Similarly, in our experience, we have found the duration^-1^ notation for rates to be more intelligible to clinical and decision-making audiences and have opted to retain it.

We have also noted in the supplemental tables that the admission and discharges are not true transitions, with a reference to the appropriate source.
